# Effects of Flavonoids from *Potamogeton crispus* L. on Proliferation, Migration, and Invasion of Human Ovarian Cancer Cells

**DOI:** 10.1371/journal.pone.0130685

**Published:** 2015-06-22

**Authors:** Yuanda Du, Jinhong Feng, Renqing Wang, Haijie Zhang, Jian Liu

**Affiliations:** 1 Institute of Environmental Research, Shandong University, Jinan, 250100, China; 2 Shandong Analysis and Test Center, Shandong Academy of Sciences, Jinan, 250014, China; 3 School of Life Sciences, Shandong University, Jinan, 250100, China; 4 Shandong Provincial Engineering and Technology Research Center for Vegetation Ecology, Shandong University, Jinan, 250100, China; Seoul National University, KOREA, REPUBLIC OF

## Abstract

In order to explore the efficient utilization of plant resources from constructed wetlands, the potential anti-metastatic effects of flavonoids from *Potamogeton crispus *L. were investigated in human ovarian cancer cells (ES-2). Two major flavonoids, luteolin-3ʹ-O-β-D-glucopyranoside and flavone-6-C-β-D-glucopyranoside, were isolated from *P*. *crispus* and identified. The effects of these flavonoids on cell proliferation, cell morphology, cell cycle, apoptosis, and cell migration and invasion were then investigated. Furthermore, reverse transcriptase polymerase chain reaction assays and western blotting analysis were conducted to examine the expression level of mRNA and protein. Results indicated that Luteolin-3ʹ-O-β-D-glucopyranoside inhibited ES-2 cell migration and invasion and suppressed the expression of two matrix metalloproteinases (MMPs), MMP-2 and MMP-9, and Flavone-6-C-β-D-glucopyranoside had no significant inhibitory effects on ES-2 cells. Thus, this study demonstrated the potential anti-metastatic properties of a *P*. *crispus* flavonoid, and provided a scientific approach for the screening of promising natural resources from constructed wetlands to identify useful products for use in the pharmaceutical and healthcare industries.

## Introduction

Plants play a key role in the construction of engineered wetland environments and they should be managed strictly to maintain wetland efficiency while minimizing the risk of secondary pollution and negative ecological effects on the ecosystem. Efficient utilization of high-biomass wetland plant resources is important because it encourages harvesting and sustainable management of constructed wetlands. *Potamogeton crispus* L. is one of the most important plants employed in constructed wetland ecosystems [1]. Previous reports have identified carotenoids, fatty acids, lignan, labdane diterpenoids, flavonoids, and phytosterins in *P*. *crispus* [2–5], and carotenoid extracts from *P*. *crispus* were reported to induce apoptosis in HeLa cells *in vitro* [6]. Our preliminary study showed anti-tumor activities of a *P*. *crispus* extract in human breast and ovarian cancer cell lines [7], and chemical analyses suggested flavonoids to be the main constituents of this extract.

It is well established that flavonoids have a large range of biochemical activities and they play an important role in the human healthcare industry [8–9]. Epidemiological and clinical data indicate that dietary flavonoids make crucial contributions to the prevention and/or management of chronic diseases such as cancer, diabetes, cardiovascular diseases and human immunodeficiency virus infection, [10–14]. Recent research on flavonoid properties has been focused on their cytotoxic antitumor activities, and experimental studies have indicated that flavonoids suppress migration and invasion, affect cell cycle progression, and induce apoptosis in several tumor cell lines [15–16].

Cancer metastasis is the leading cause of mortality in patients with malignant tumors, and is estimated to be responsible for 90% of human cancer-related deaths [17]; it thus remains an important challenge for cancer therapy. Degradation of the extracellular matrix (ECM) is a crucial feature of metastatic tumors and this process is associated with the over-expression of matrix metalloproteinases (MMPs) [18–19]. It has been reported that luteolin and baicalein flavonoids inhibit metastasis by suppressing the expression and secretion of MMP2 and MMP9 in human breast cancer cells (MCF-7 and MDA-MB-231) and in hepatocellular carcinoma cells (MHCC97H) [20–22]. However, it is unclear whether flavonoids have anti-metastatic effects on ovarian cancer cells.

In the present study, we purified two flavonoids from *P*. *crispus* and examined their effects on the human ovarian cancer ES-2 cell line. The proliferation, morphology, cell cycle progression, apoptosis, migration, and invasion of these cells were investigated with the aim of elucidating the effects of *P*. *crispus* flavonoids on ES-2 cells and the mechanisms involved.

## Materials and Methods

### Ethics statement

The field survey and sample collection involved in this study were conducted with the official permission of the Environmental Protection Bureau of Weishan County and the Management Committee of Xinxue River constructed wetland. The fieldwork did not involve any endangered or protected plant species or any animal species. The laboratory protocol was approved by the Shandong University ethics committee.

### Preparation of plant material

*P*. *crispus* material was collected in the Xinxue River constructed wetland (117.16°E, 34.78°N), at Nansi Lake, Weishan county, China. The collection was conducted in early July, when *P*. *crispus* had the maximum biomass. The whole plant was dried, powdered, and extracted with ethanol under heating reflux three times, for 90 min per extraction. The ethanol extract was then suspended in water before partitioning with petroleum ether (PE), ethyl acetate (EtOAc), and n-butanol sequentially; these were concentrated under a vacuum to give a PE extract, an EtOAc extract, and an n-butanol extract. Based on our previous studies [7], the EtOAc extract was selected for further separation. The EtOAc extract was chromatographed on an MCI gel column, followed by Sephadex LH-20 column chromatography, the two main compounds were then prepared using high-performance liquid chromatography (HPLC) (Agilent 6270, USA). The two compounds were identified by HPLC, nuclear magnetic resonance (NMR) (AVANCE 600, Bruker, Germany), and high-resolution electrospray ionization mass spectrometry (HR-ESI-MS) (LTQ Orbitrap XL, ThermoFisher, USA).

### Cell culture and treatment

The ES-2 human ovarian cancer cell line was obtained from the Shandong Analysis and Test Center in August 2014. Cells were cultured in RPMI-1640 medium (HyClone, USA) supplemented with 10% fetal bovine serum (FBS) (Gibco, Life Technologies, USA) and antibiotics (100 μg/mL penicillin and 100 μg/mL streptomycin) (HyClone). Cell cultures were maintained at 37°C in a humidified atmosphere with 5% CO_2_. Compounds were dissolved at a concentration of 0.1 M in 100% dimethyl sulfoxide (DMSO) (Solarbio, China) to form stock solutions, stored at -20°C, and diluted to the indicated concentrations with the medium before each experiment. The final DMSO concentration did not exceed 0.1% throughout the study, and all the control groups were exposed to 0.1% DMSO.

### Cell proliferation assay

Flavonoid effects on cell proliferation were evaluated by using the MTT (thiazolyl blue tetrazolium bromide) (Solarbio) assay. ES-2 cells were seeded (1 × 10^4^ per well) on a 96-well plate in RPMI-1640 supplemented with 10% FBS. After overnight incubation at 37°C in 5% CO_2_, different concentrations (15–240 μg/mL) of the compounds were added into triplicate wells. After incubation for 48 h, MTT was added to each well to achieve a final concentration of 0.5 mg/mL and incubation was performed for an additional 4 h. The medium was then replaced with the stop solution (DMSO; 150 μL per well) and the absorbance at 490 nm was measured on a plate reader (EnSpire, Perkin Elmer Corporation, USA). The 0.1% DMSO controls were measured in parallel. Cytotoxicity was expressed as the inhibition rate, which was calculated as follows:

$$\left(A_{control}-A_{sample}/A_{control}-A_{0}\right)\;\times \;100\%$$

where A_control_ = the absorbance of control wells; A_sample_ = the absorbance of treated wells; A_0_ = the absorbance of blank wells. Origin 7.5 software was used to analyze the data and calculate IC_50_ values. Assays were repeated at least three times.

### Observation of cell morphology

ES-2 cells were cultured as described for the cell proliferation assay. The cells were treated with 30 or 60 μg/mL luteolin-3′-O-β-d-glucopyranoside (LU3′O-GP) or 100 μg/mL flavone-6-C-β-d-glucopyranoside (FL6C-GP). Cell morphology observations were carried out after incubation for 48 h, using an inverted fluorescent microscope (Ti-S, Nikon, Japan).

### Cell cycle progression assay

ES-2 cells (5 × 10^4^ per well) were seeded on a 6-well plate and incubated overnight at 37°C in 5% CO_2_ prior to addition of the indicated compounds prepared in complete media for 48 h. The cells were then collected by trypsinization, washed in phosphate-buffered saline (PBS), and fixed in 70% ethanol at 4°C for 2 h. The cells were washed twice with PBS, and centrifuged at 2000 rpm for 5 min; the supernatant was discarded. The cells were re-suspended in 100 μL of PBS containing RNAse (50 μg/mL) and incubated at 37°C for 30 min; the reaction was then stopped by placing on ice for 2 min. ES-2 cells were stained by incubation with 10 μg/mL propidium iodide (PI) with 0.1% Triton X-100 in the dark at 4°C for 30 min. The samples were analyzed by flow cytometry (FACSAria, BD, USA) and the results were analyzed by FlowJo 7.6.1.

### Cell apoptosis assay

The annexin-V-PE/7-AAD apoptosis detection kit (BD Pharmingen, USA) was used to investigate the effects of the test compounds on apoptosis in ES-2 cells. Annexin V is a protein that has a high affinity for phosphatidylserine (PS). During apoptosis, PS translocates from the inner face of the plasma membrane to the cell surface, where it can be detected using a fluorescent annexin V conjugate. ES-2 cells were cultured and seeded as described above for the cell cycle progression assay. After incubation with *P*. *crispus* flavonoids for 48 h, cells were collected by trypsinization, washed twice with PBS, and re-suspended in 400 μL of binding buffer prior to the addition of 5 μL of annexin V-PE, incubation in the dark at 4°C for 15 min, and staining with 10 μL of 7-AAD solution. The samples were analyzed by flow cytometry (FACSAria, BD) after incubation in the dark at 4°C for 15 min, and the results were analyzed by FlowJo 7.6.1 software. Apoptotic and necrotic cells were identified by annexin V positive/7-AAD negative staining and annexin V positive/7-AAD positive staining, respectively.

### Cell migration assay

Cell migration assays were carried out using Transwell chambers (Corning Costar, USA) The Transwell chamber was filled with medium and 0.1% bovine serum albumin (BSA) (Gibco, Life Technologies)was included in the serum-free medium in the upper chamber, to maintain the osmotic pressure. ES-2 cells were exposed to the indicated flavonoid concentrations in a serum-free medium for 48 h prior to adding the cells (2 × 10^5^) to the upper Transwell chamber. The bottom chamber contained medium with 5% FBS to serve as a chemo-attractant. The chambers were incubated for 24 h and the non-migration cells on the upper side of the Transwell membrane were removed using cotton swabs. The membrane was fixed in 100% methanol; the migrated cells were then stained with 0.1% crystal violet for 10 min, and washed three times with PBS. The membrane was photographed under a microscope before being washed with 33% acetic acid; the absorbance of the eluent was measured at 590 nm in a plate reader (EnSpire, Perkin Elmer Corporation).

### Cell invasion assay

Cell invasion was assayed using the protocol described above for the cell migration assay, except that the Transwell chamber was coated with Matrigel (BD Pharmingen) and the medium was free of BSA.

### Isolation of total RNA and reverse transcriptase polymerase chain reaction (RT-PCR) assay

ES-2 cells were seeded in a 6-well plate (4 × 10^5^ cells per well) and incubated overnight before treatment with various concentrations of LU3′O-GP (0, 30, 60, 90 μg/mL) for 48 h. Cells were collected by trypsinization and washed in PBS. Total RNA was isolated using Trizol (Invitrogen, Life Technologies) and 5 mg was used to synthesize cDNA using M-MLV (Invitrogen, Life Technologies). The cDNA was amplified using the following primer sequences (BGI, China):

MMP2, forward, 5′-GTTCATTTGGCGGACTGT-3′, reverse, 5′-AGGGTGCTGGCTGAGTAG-3′;

MMP9, forward, 5′-AATCTCACCGACAGGCAGCT-3′, reverse, 5′-CCAAACTGGATGACGATGTC-3′;

Glyceraldehyde 3-phosphate dehydrogenase (GAPDH), forward, 5′-GAAGGTGAAGGTCGGAGT-3′, reverse, 5′-CATGGGTGGAATCATATTGGAA-3′.

PCR was performed using the following program: 95°C for 5 min; then 45 cycles of 95°C for 10 s; 55°C for 15 s; and 72°C for 10 s. The samples were then heated (72°C for 10 min) and cooled to 4°C. GAPDH was used as the RNA loading control. The PCR products were separated on 1% agarose gels and detected by ethidium bromide staining. The results were analyzed by BandScan5.0.

### Western blotting analysis

ES-2 cells were seeded in a 6-well plate (4 × 10^5^ cells per well) and incubated overnight before treatment with various concentrations of LU3′O-GP (0, 30, 60, 90 μg/ml) for 48 h. Cells were suspended in 500 μL of lysis buffer at 4°C (40 mM Tris-HCl, 1 mM EDTA, 150 mM KCl, 100 mM NaVO3, 1% Triton X-100, 1 mM PMSF, pH 7.5). The proteins were separated by 10% sodium dodecyl sulfate-polyacrylamide gel electrophoresis and transferred onto PVDF membranes. The membranes were subsequently blocked using skimmed milk in 5% Tris-buffered saline with Tween-20 (TBST) at 37°C for 1 h and incubated overnight with primary antibodies (MMP-2 antibody: Santa Cruz Biotechnology, USA; MMP-9 antibody: RabMab, abcam, UK) in TBST containing 5% skimmed milk at 4°C. The membranes were then incubated with a secondary antibody for 1 h at room temperature. The bands were detected by enhanced chemiluminescence and photographed; the results were analyzed by Image J software.

### Statistical analysis

For each assay, triplicate experiments were performed, and the results are presented as the mean ± standard deviation (SD). The significance of group differences was examined using Student’s two-tailed t-test (Microsoft Excel software).

## Results

### Identification of two main *P*. *crispus* flavonoids

**Fig 1:**
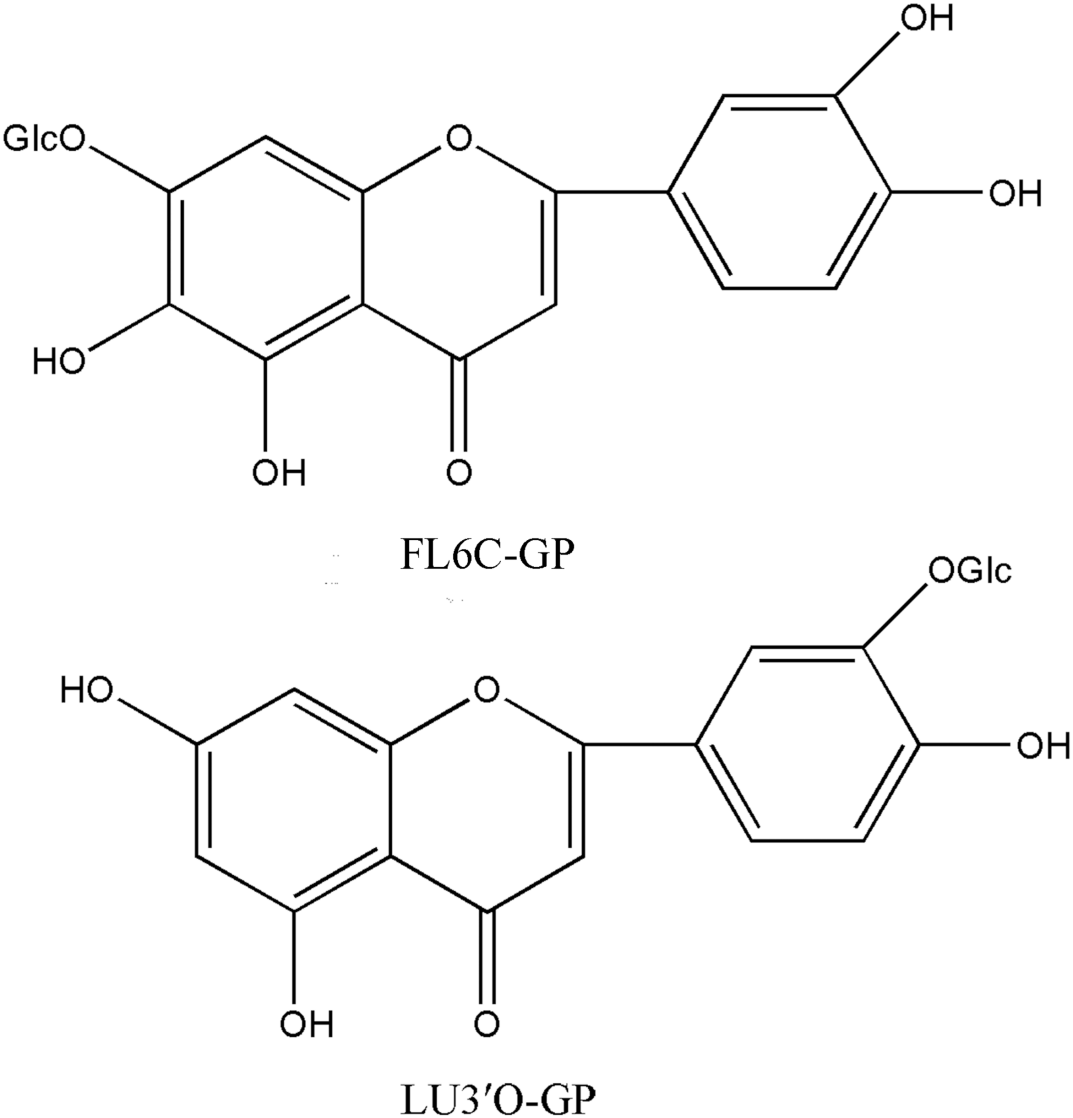
Chemical structure of two *P*. *crispus* flavonoids

HPLC, NMR, and HR-ESI-MS were conducted to isolate and identify the two main compounds within the *P*. *crispus* EtOAc extract. These compounds were identified and characterized as Luteolin-3′-O-β-d-glucopyranoside (LU3′O-GP) and Flavone-6-C-β-d-glucopyranoside (FL6C-GP) by comparing their spectral data (HPLC, HR-ESI-MS, ^1^H and ^13^C NMR) and physicochemical properties with those reported in the literature [23–25]. This is the first report of extraction of these two flavonoids from *P*. *crispus*. The molecular structure of two compounds were shown in Fig 1, and the purity of both the compounds was above 90% (Figs a and b in S1 File).

### LU3′O-GP inhibited ES-2 cell proliferation

**Fig 2:**
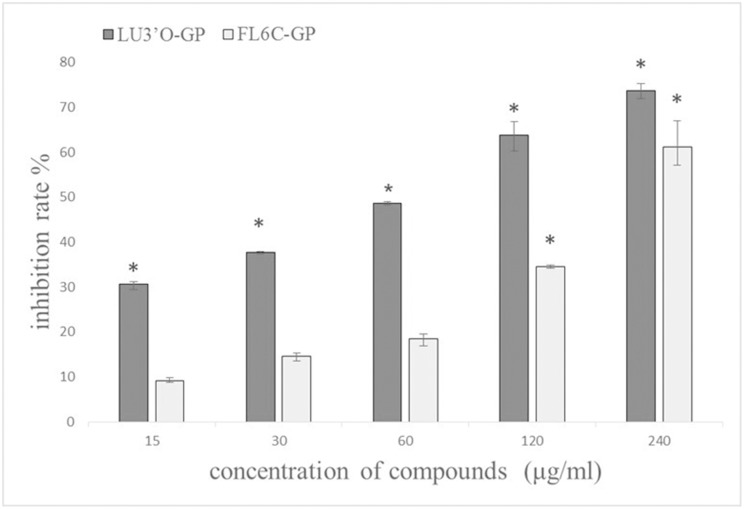
Proliferation inhibition of two *P*. *crispus* flavonoids on ES-2 cells. Cells were incubated with five different flavonoid concentrations or dimethyl sulfoxide (DMSO; control) for 48 h. Data represent the means of triplicate experiments ± standard deviation (SD) * *p* < 0.01.

ES-2 ovarian cancer cells were incubated for 48 h with five different concentrations of LU3′O-GP or FL6C-GP. The MTT assay results indicated a concentration-dependent decrease in cell viability in the presence of LU3′O-GP (Fig 2). Cell viability decreased by 63.8% and 73.6% after 48 h exposure to 120 and 240 μg/mL LU3′O-GP, respectively, and the IC_50_ value was 57.1 μg/mL. The same concentrations of FL6C-GP had a significantly lower impact on cell viability and this compound had an IC_50_ value of 182.7 μg/mL. These results indicated that LU3′O-GP inhibited the proliferation of ES-2 cells in a concentration-dependent manner.

### LU3′O-GP affected ES-2 morphology

**Fig 3:**
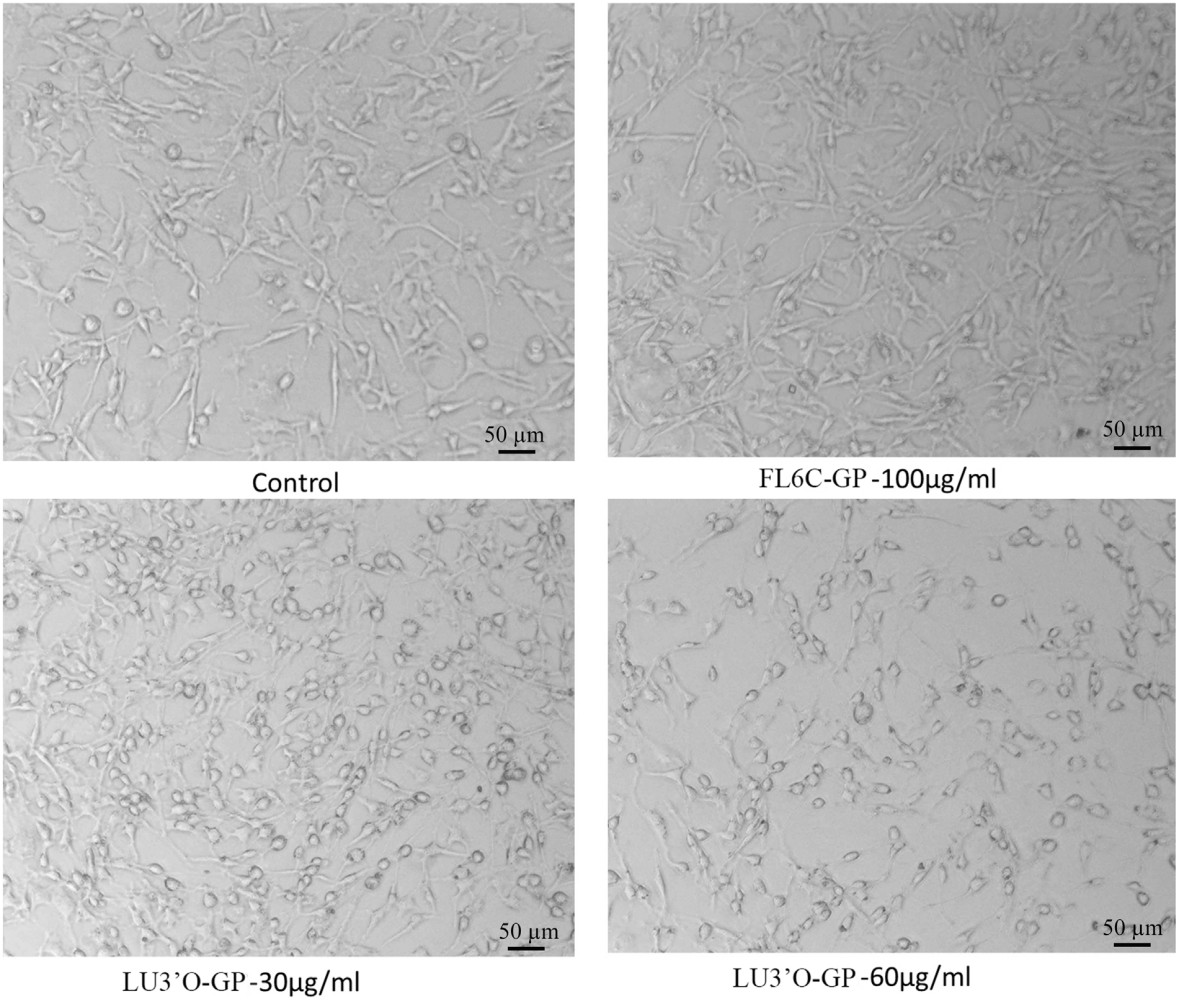
ES-2 cell morphology. ES-2 cells were treated with the indicated *P*. *crispus* flavonoids for 48 h, and imaged using an inverted fluorescent microscope.

Morphological examinations were conducted to further investigate the effect of LU3′O-GP on ES-2 cells. The ES-2 cells were treated with concentrations of LU3′O-GP associated with low cytotoxicity (30 and 60 μg/mL), or 100 μg/mL FL6C-GP. Images obtained by inverted microscopy after 48-h incubations are shown in Fig 3. The control and FL6C-GP comparison cells showed the classical spindle cell pattern observed in ovarian tumor cells. The cells treated with LU3′O-GP showed a significant loss of mobility and changes in cell morphology; the major morphological change was to the cytomere shape, which became spherical, with a shortened tentacle. These changes occurred in a concentration-dependent manner. The FL6C-GP treatment produced no significant change, as compared with control cells. These results indicated that LU3′O-GP could change the cellular morphology of ES-2 cells and suggested its potential influence on the cell mobility.

### LU3′O-GP induced cell cycle arrest in ES-2 cells

**Fig 4:**
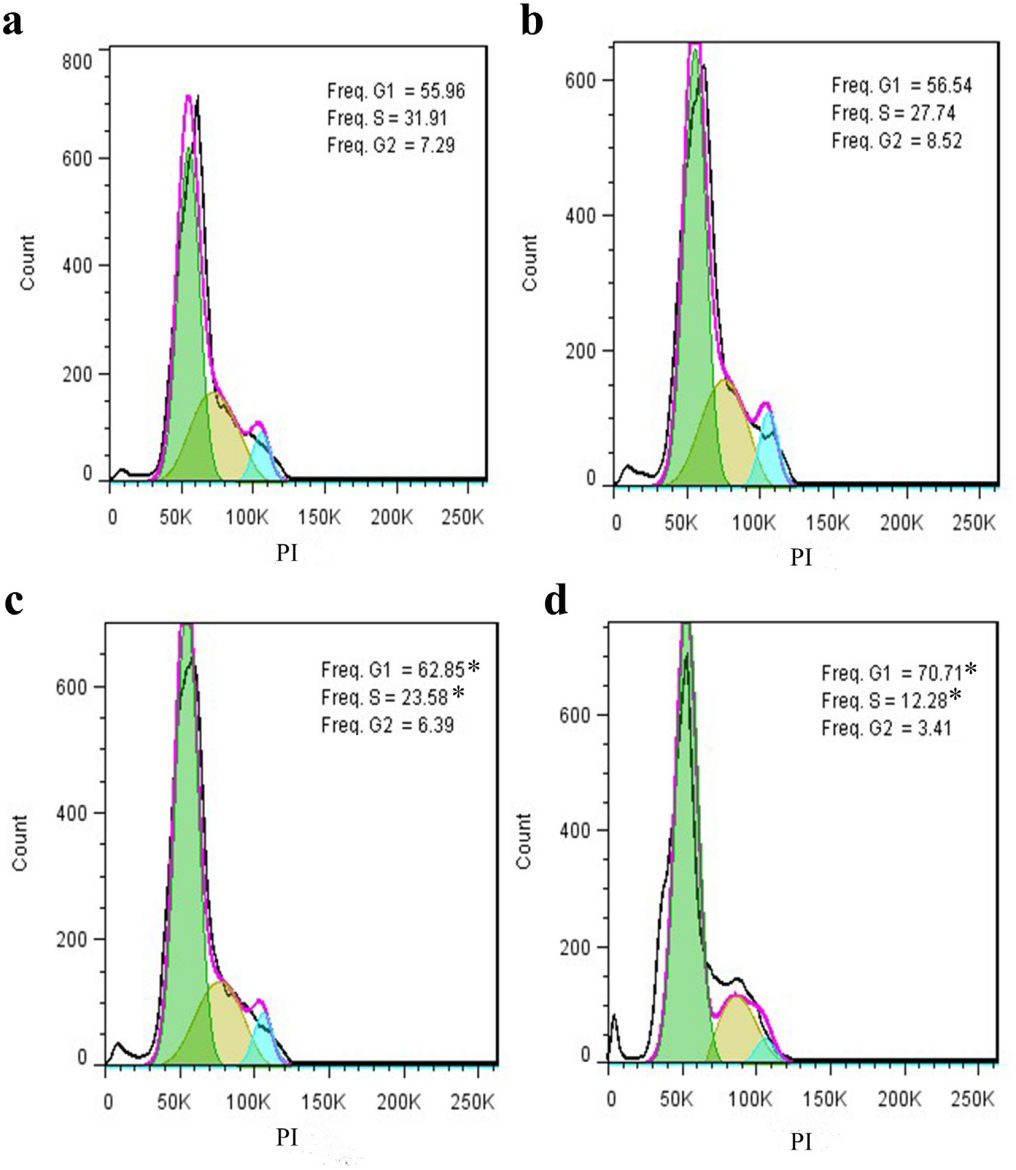
*P*. *crispus* flavonoid effects on cell cycle progression in ES-2 cells. (a) Negative control; (b) cells treated with 100 μg/mL flavone-6-C-β-d-glucopyranoside (FL6C-GP); (c) cells treated with 30 μg/mL luteolin-3′-O-β-d-glucopyranoside (LU3′O-GP); and (d) cells treated with 60 μg/mL LU3′O-GP. Cells were fixed with ethanol and stained with propidium iodide prior to cell cycle distribution analysis by flow cytometry. The number of cells in G0/G1 phase is represented by the first peak, and those in G2/M phase are represented by the second peak. Cells in S phase are present in the area between the G0/G1 and G2/M peaks. These data represent the mean of triplicate experiments. * *p <* 0.01 as compared with control.

To determine whether *P*. *crispus* flavonoids affected the cell cycle, ES-2 cells treated with either LU3′O-GP or 100 μg/mL of FL6C-GP for comparison, prior to analysis by flow cytometry. The results (Fig 4) indicated that LU3′O-GP treatment caused concentration-dependent increases in the number of cells in G0/G1 phase, and decreases in the number of cells in S phase, while FL6C-GP treatment had no significant effects on cell cycle progression in ES-2 cells. These results demonstrated that LU3′O-GP affected ES-2 cell cycle progression by causing cell cycle arrest at the phase boundary of G1 phase and S phase.

### *P*. *crispus* flavonoids had no effect on apoptosis in ES-2 cells

**Fig 5:**
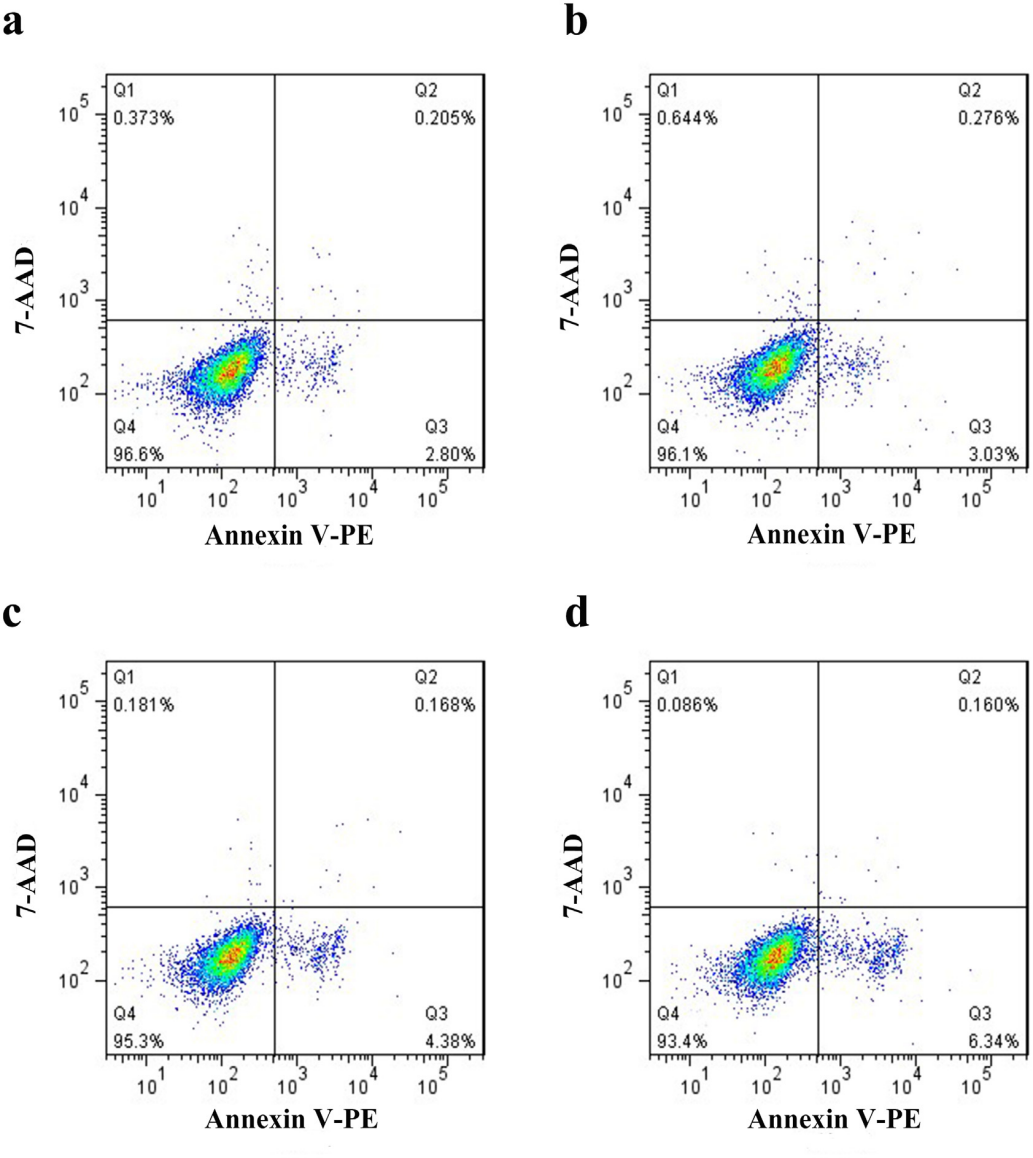
Quantification of apoptotic ES-2 cells after treatment with *P*. *crispus* flavonoids. (a) Negative control; (b) cells treated with 100 μg/mL flavone-6-C-β-d-glucopyranoside (FL6C-GP); (c) cells treated with 30 μg/mL luteolin-3′-O-β-d-glucopyranoside (LU3′O-GP); (d) cells treated with 60 μg/mL LU3′O-GP. Apoptotic cells are shown in Q3.

Apoptosis is an active and physiological mode of cell death that is commonly restored by anti-cancer agents. Flow cytometry was used to determine whether LU3′O-GP affected ES-2 apoptosis. As depicted in Fig 5, the cell population in the Q3 zone represented the apoptotic cells, which were annexin V positive and 7-AAD negative. This population showed no obvious change in the presence of LU3′O-GP or FL6C-GP, suggesting that *P*. *crispus* flavonoids had no effect on apoptosis in ES-2 cells.

### LU3′O-GP inhibited migration and invasion of ES-2 cells

**Fig 6:**
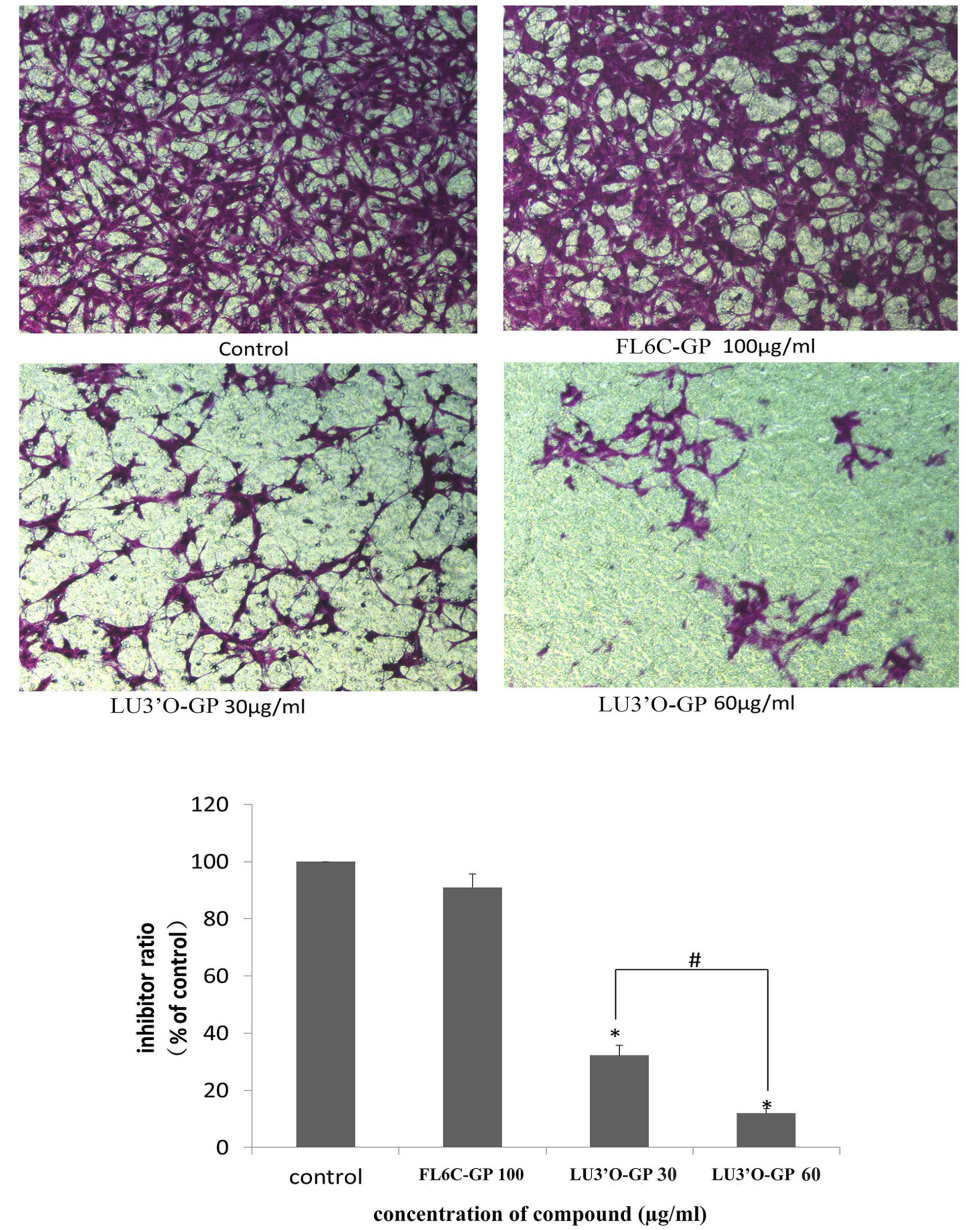
Effects of *P*. *crispus* flavonoids on ES-2 cell migration. Cells were exposed to the indicated concentrations of luteolin-3′-O-β-d-glucopyranoside (LU3′O-GP) and flavone-6-C-β-d-glucopyranoside (FL6C-GP), and their migration was quantified using a Transwell chamber. Values represent the means of triplicate experiments. *p < 0.01, as compared to the control; #*p* < 0.05, as compared with the indicated concentration.

Cell motility is considered to be an important determinant of the metastatic potential of cancer cells. The effect of LU3′O-GP on the motility of ES-2 cancer cells was examined using a cell migration assay. As shown in Fig 6, the migration of ES-2 cells was significantly decreased in a concentration-dependent manner after exposure to LU3′O-GP. This inhibition was about 67.7% and 88.1%, as compared with control cells, in the presence of 30 and 60 μg/mL LU3′O-GP, respectively. The cells treated with FL6C-GP showed no significant change in migration activity, as compared with control cells.

**Fig 7:**
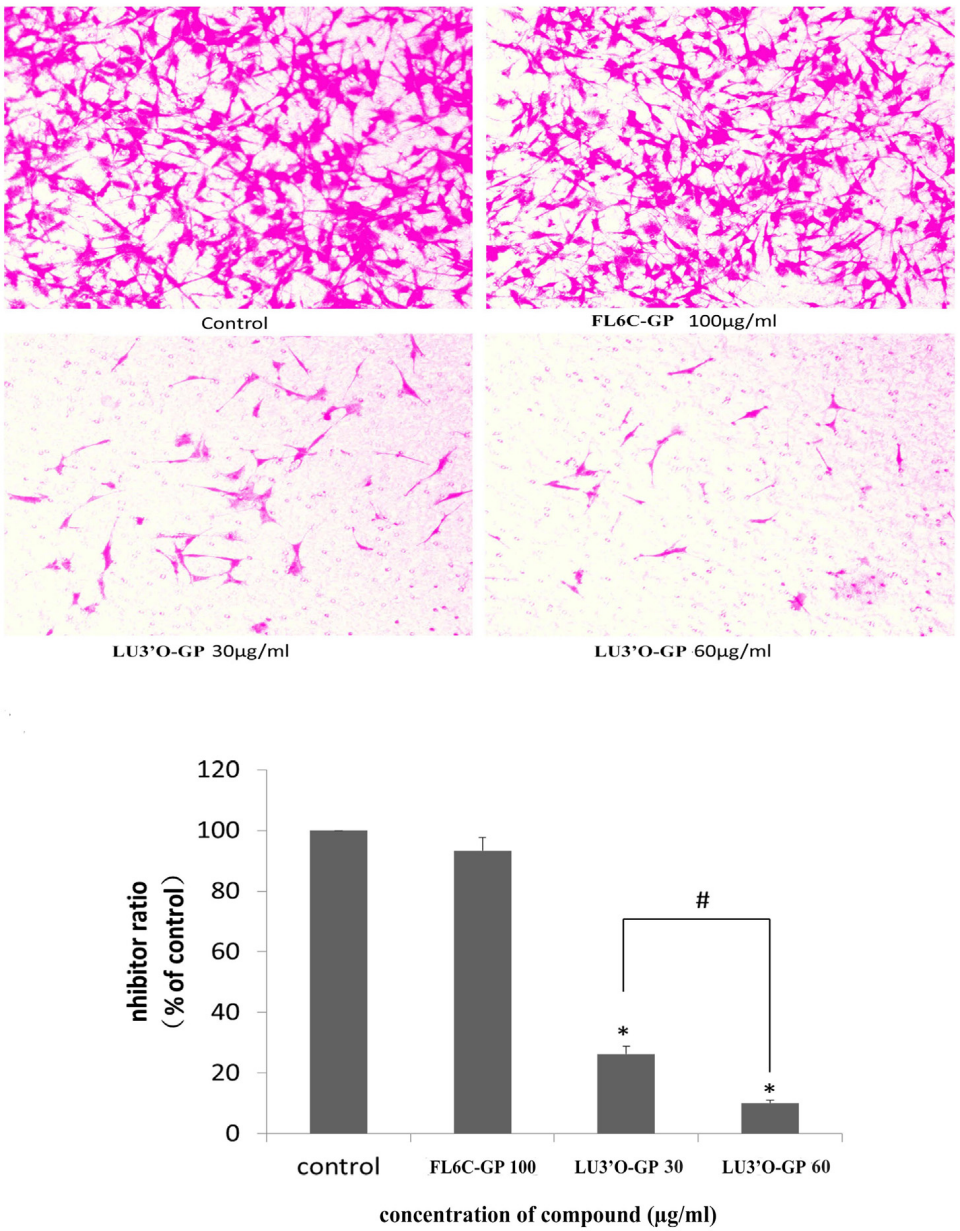
Effects of *P*. *crispus* flavonoids on ES-2 cell invasion. Cells were treated with luteolin-3′-O-β-d-glucopyranoside (LU3′O-GP) or flavone-6-C-β-d-glucopyranoside (FL6C-GP), and their invasion was quantified in a Transwell chamber membrane with Matrigel. Values represent the means of experiments performed in triplicate. *p < 0.01, as compared to the control; #*p* < 0.05, as compared with the indicated concentration.

During tumor metastasis, the cells must pass through the ECM. To evaluate whether LU3′O-GP affected ES-2 cell invasion, cell invasion assays were carried out in a Transwell chamber coated with Matrigel. Treatment of ES-2 cells with increasing concentrations of LU3′O-GP led to a significant concentration-dependent decrease in the cell invasion rate, as compared with the control (Fig 7). This process was inhibited by about 73.7% and 89.9% in the presence of 30 and 60 μg/mL LU3′O-GP, respectively, while the inhibition induced by 100 μg/mL FL6C-GP was only 6.7%. The results of this assay demonstrated that LU3′O-GP dramatically inhibited the invasion of ES-2 cells.

**Fig 8:**
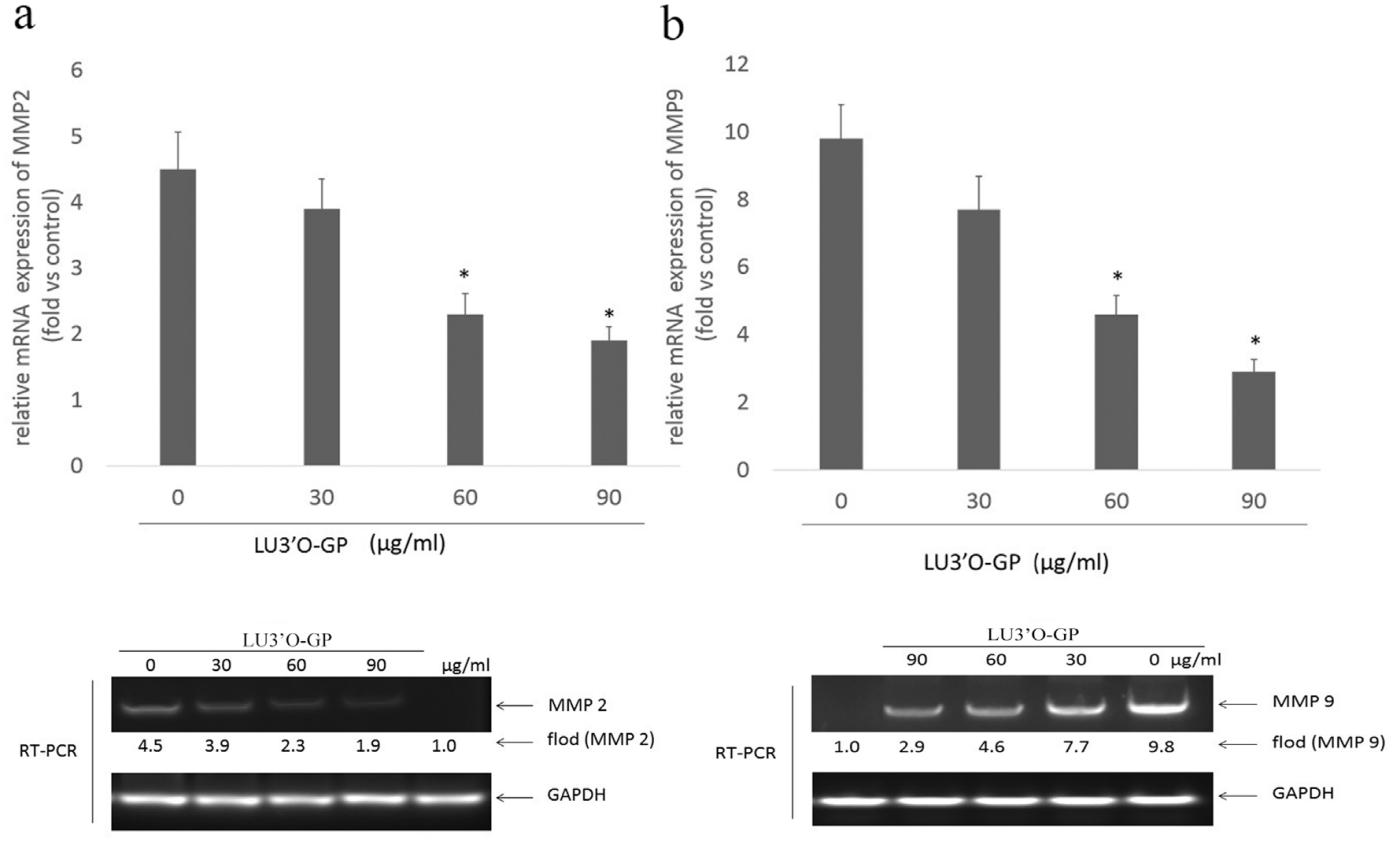
Luteolin-3′-O-β-d-glucopyranoside (LU3′O-GP) reduced mRNA expression of MMP-2 and MMP-9 in ES-2 cells. (a) LU3′O-GP effects on MMP-2 expression; (b) LU3′O-GP effects on MMP-9 expression. mRNA levels were investigated by reverse transcriptase polymerase chain reaction (RT-PCR), using glyceraldehyde 3-phosphate dehydrogenase as the loading control. RT-PCR products were detected by ethidium bromide staining. Values represent the means of triplicate experiments. *p < 0.05, as compared to the control.

**Fig 9:**
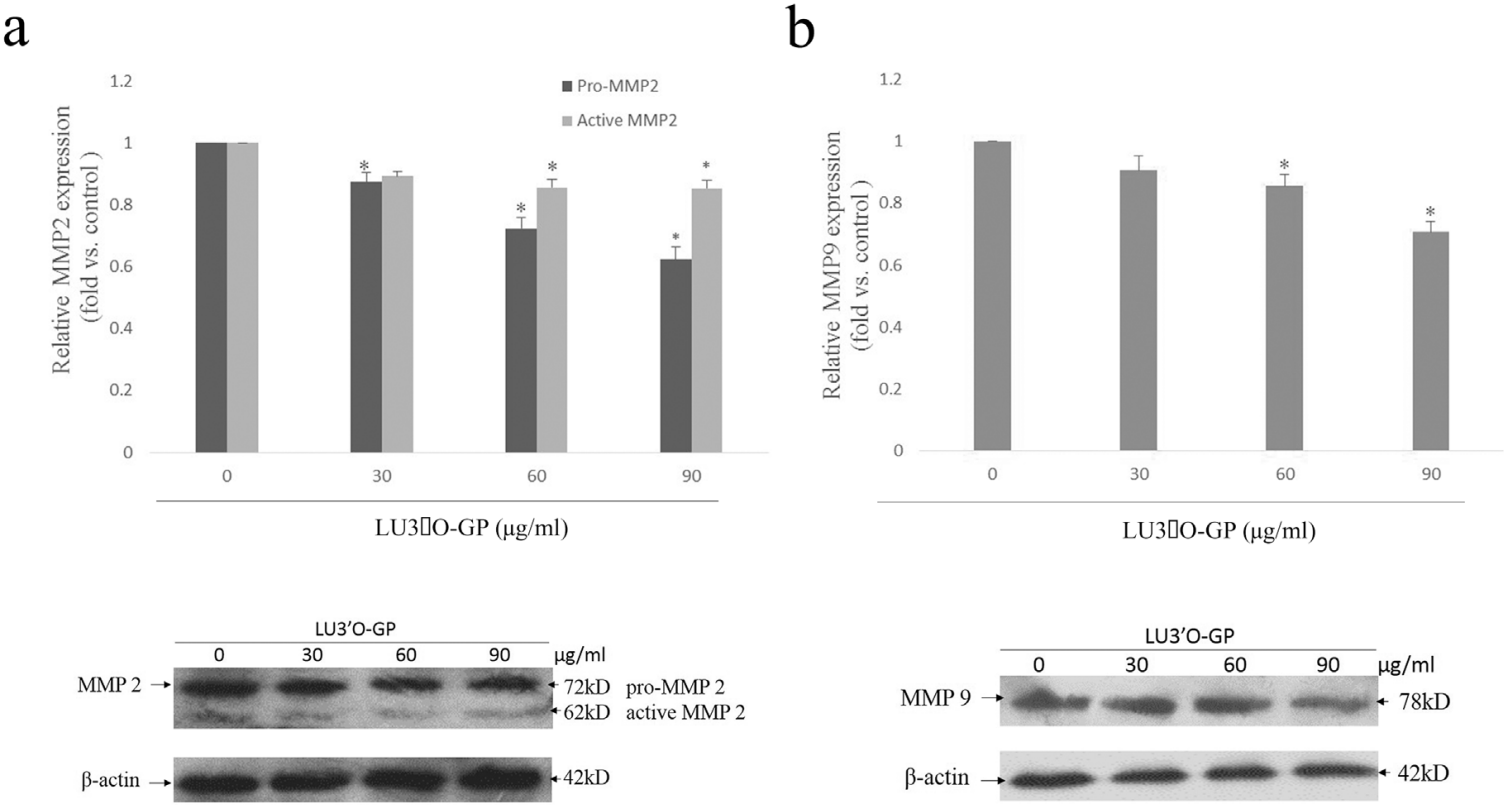
Luteolin-3′-O-β-d-glucopyranoside (LU3′O-GP) reduced protein expression of MMP-2 and MMP-9 of in ES-2 cells. (a) LU3′O-GP effects on MMP-2(pro-MMP-2 and activity MMP-2); (b) LU3′O-GP effects on MMP-9 expression. Protein levels were investigated by western boltting analysis. Values represent the means of triplicate experiments. *p < 0.05, as compared to the control.

To further determine the influence of LU3′O-GP on the upstream factors that are important for the regulation of tumor cell invasion, the mRNA and protein expression of MMP2 and MMP9 were investigated. The RT-PCR assay results are shown in Fig 8. The levels of MMP2 and MMP9 mRNAs were reduced in a concentration-dependent manner by treatment with LU3′O-GP, Western blotting analysis (Fig 9) showed a similar suppression of MMP-2 (pro-MMP2 and activity MMP-2) and MMP-9 protein expression by LU3′O-GP. These results suggested that the flavonoid, LU3′O-GP, suppressed expression of MMP2 and MMP9 at both the mRNA and protein levels.

## Discussion

In this study, two major flavonoids, LU3′O-GP and FL6C-GP, were isolated and identified from *P*. *crispus* with the aim of exploring the potential anti-metastatic effects of this constructed wetland plant resource. Metastasis is the major cause of death in cancer patients and the development of new treatment regimens that inhibit tumor dissemination is crucial for successful cancer therapy and prevention. However, most highly cytotoxic synthetic anti-cancer drugs possess a low specificity; this produces effects on normal tissues, in addition to tumor cells, leading to poor clinical outcomes. Considerable emphasis has therefore been given to the identification of new anti-cancer agents from natural sources, and edible resources are particularly valuable because of their high margin of safety; many natural dietary agents are currently in early phase clinical trials [26]. Flavonoids provide one of the most outstanding natural resources in this context. Dietary flavonoids almost all exist as glycosides in nature and the most abundant plant flavonoid glycosides are flavone O/C-glycosides and flavonol O-glycosides [27].

LU3′O-GP is a flavone O-glycoside and FL6C-GP is a flavone C-glycoside. However, the present study indicated that LU3′O-GP produced significant effects on ES-2 proliferation, morphology, cell cycle progression, migration, and invasion, whereas FL6C-GP had no apparent effects on these cells. We hypothesized that this difference was related to two differences between these compounds. Firstly, flavonoid aglycones may show different bioactivities. Luteolin, the aglycones of LU3′O-GP, and some luteolin C-glycosides have been demonstrated to inhibit tumor cell survival and metastasis in various human epithelioid cancer cells including MCF-7, MDA-MB231, and LNM35 cells [21, 28–29]. Secondly, the flavonoid glycosides are too water-soluble to diffuse across the cell membrane, whereas the aglycones are more hydrophobic and can easily enter cells by passive diffusion; deglycosylation of flavonoid glycosides to their aglycones is therefore considered to be the first stage of metabolism, producing effects *in vivo* [30]. Therefore, different sugar moieties bound to flavonoid aglycones could influence their deglycosylation and metabolism to a certain degree and result in differences in bioactivity [31]. In the present study, LU3′O-GP did not induce apoptosis in ES-2 cells, although luteolin was previously reported to induce apoptosis in some human cancer cells and in mouse neuroblastoma cells [32–33]. Thus, our results may also provide evidence for flavonoid glycosylation-related variation in activity.

However, it is possible that the effects of glycosylation on flavonoid bioactivity *in vitr*o may differ from those observed *in vivo*. Flavonoid glycosides have been reported to show similar or even greater anti-diabetic, anti-inflammatory, anti-degranulating, anti-stress, and anti-allergy activities than their flavonoid aglycones *in vivo* [31]. Overall, it is very difficult to draw general conclusions regarding the impact of glycosylation on flavonoid bioactivities and further research is needed to understand the relationship between flavonoid glycosylation and bioactivity *in vivo* and *in vitro*.

MMP-2 and MMP-9 are crucial enzymes that are considered to be important contributors to the processes of invasive metastasis and angiogenesis in various tumors [34–37]. MMP-9 and MMP-2 expression levels are significantly elevated in a range of carcinomas [36, 38]. Therefore, inhibition of MMP-2 and MMP-9 expression should be a high priority during cancer therapy. In the present study, ES-2 cells were treated with LU3′O-GP at concentrations associated with low cytotoxicity in order to evaluate the anti-metastatic activity of this compound, and an apoptosis assay was also conducted. These findings indicated that LU3′O-GP significantly inhibited ES-2 cell migration and invasion and these effects were closely related to a suppressed expression of MMP-2 and MMP-9 in mRNA and protein level in the absence of cytotoxic or apoptotic effects. Additionally, given that the metastatic spread of tumor cells is a multi-stage process involving a series of complex physiological events, MMP-2 and MMP-9 expression is regulated by a complicated network of signaling pathways that can be activated by a range of growth factors, cytokines, and chemicals such as PI3K/Akt, p38-MAPK, NF-κB, EGFR, ERK1/2, and TPA, acting via a number of pathways, such as the MAPK/ERK pathway [39–45]. This indicates that the mechanisms underlying metastasis may be specific to different types of tumor cell. The clear LU3′O-GP-induced inhibition of ES-2 metastasis should therefore be explored in other types of tumor cell.

*P*. *crispus* is a widely used species in constructed wetlands and it should be harvested at the appropriate time to maintain effective pollutant removal and to avoid secondary pollution and negative ecological effects. The large amount of *P*. *crispus* biomass can make post-harvest procedures challenging. Natural products and herbal medicines are promising sources of new therapeutic agents for the human healthcare industry [46]. In this study, flavonoids with anti-metastatic activity were isolated from *P*. *crispus*, indicating that this species had potential health benefits. Our study provided a scientific basis for the screening of promising natural resources as sources of medicines and suggested a potential approach to the efficient and sustainable utilization of plant resources in constructed wetlands.

## Supporting Information

S1 File High-performance liquid chromatography (HPLC) analysis of two flavonoids isolated from *P*. *crispus*. Luteolin-3′-O-β-D-glucopyranoside (LU3′O-GP) (Fig a). flavone-6-C-β-D-glucopyranoside (FL6C-GP) (Fig b). The HPLC employed an Agela C18 column (symmetry R 4.6 × 250 mm) with methanol and H_2_O (40%:60%) as the mobile phase, at a flow-rate of 1 ml/min for 40 min and used UV/V detection.
(TIF)
