# Supplementary figures and images for: Effects of Flavonoids from Potamogeton crispus L. on Proliferation, Migration, and Invasion of Human Ovarian Cancer Cells
Source: PLoS One. 2015 Jun 22;10(6):e0130685. doi: 10.1371/journal.pone.0130685 (PMC4476667; doi:10.1371/journal.pone.0130685)

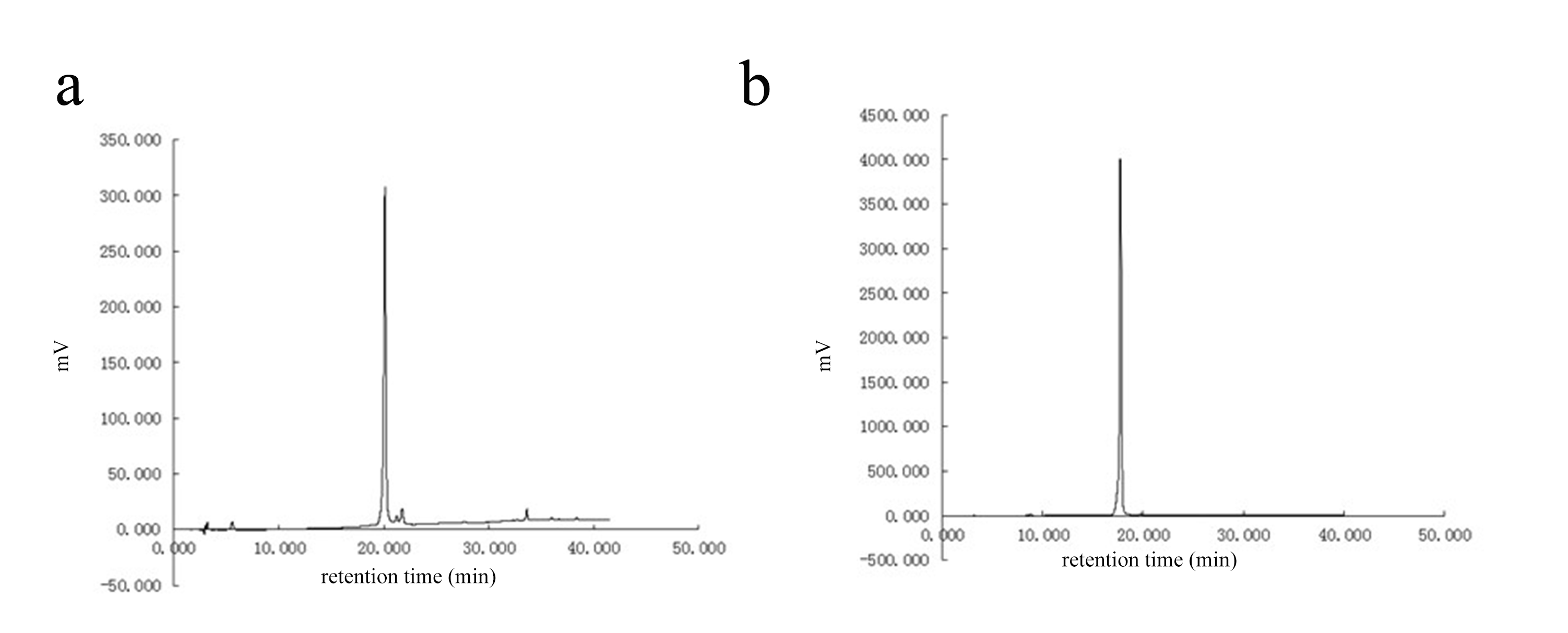

Supplement: S1 File — Luteolin-3′-O-β-D-glucopyranoside (LU3′O-GP) (Fig a). flavone-6-C-β-D-glucopyranoside (FL6C-GP) (Fig b). The HPLC employed an Agela C18 column (symmetry R 4.6 × 250 mm) with methanol and H2O (40%:60%) as the mobile phase, at a flow-rate of 1 ml/min for 40 min and used UV/V detection. (TIF) [file pone.0130685.s001.tif]
